# Propranolol reduces viability and induces apoptosis in hemangioblastoma cells from von Hippel-Lindau patients

**DOI:** 10.1186/s13023-015-0343-5

**Published:** 2015-09-22

**Authors:** Virginia Albiñana, Karina Villar Gómez de las Heras, Gemma Serrano-Heras, Tomás Segura, Ana Belén Perona-Moratalla, Mercedes Mota-Pérez, José María de Campos, Luisa María Botella

**Affiliations:** Centro de Investigaciones Biológicas, CSIC, Madrid, Spain; SSCC del Servicio de Salud de Castilla-La Mancha (SESCAM), Toledo, Spain; Unidad de Investigación, Complejo Universitario Hospital Albacete, Albacete, Spain; Centro de Investigación Biomédica en Red de Enfermedades Raras (CIBERER), Madrid, Spain; Servicio de Neurocirugía, Fundación Jiménez Díaz, Madrid, Spain

**Keywords:** von Hippel-Lindau disease (VHL), pVHL, Hypoxia inducible factor, hemangioblastoma, CNS tumors, Propranolol

## Abstract

**Background:**

Von Hippel-Lindau (VHL) disease is a rare oncological disease with an incidence of 1:36,000, and is characterized by the growth of different types of tumors: hemangioblastomas in the central nervous system (CNS) and retina, renal carcinoma, pheochromocytomas, pancreatic serous cystadenoma, and endolymphatic sac tumors. These tumors do not express VHL protein (pVHL). pVHL ubiquitinates hypoxia inducible factor (HIF) protein for degradation by the proteasome; in the absence of VHL, HIF translocates to the nucleus to activate the expression of its target genes. Targeting VHL-derived tumors with drugs that have reduced side effects is urgent to avoid repeat CNS surgeries. Recent reports have shown that propranolol, a β-blocker used for the treatment of hypertension and other cardiac and neurological diseases, is the best option for infantile hemangioma (IH). Propranolol could be an efficient treatment to control hemangioblastoma growth in VHL disease because of its antiangiogenic effects demonstrated in IH and the hypothetical impact on HIF levels.

**Methods:**

HeLa 9X (HRE) hypoxia responsive element cell line and primary hemangioblastoma-derived cells were subjected to propranolol treatment and cell viability and apoptosis were evaluated. HIF1-α and Hif-2α expression after propranolol treatment was analyzed by western blotting. Quantitative PCR was performed to study the mRNA expression of HIF target genes. Vascular endothelial growth factor (VEGF) was measured in culture supernatants by immunoassay.

**Results:**

Propranolol downregulated HIF-dependent transcription in HeLa 9XHRE cells. Under hypoxic conditions, propranolol decreased the expression of HIF target genes in hemangioblastoma cells, which stopped proliferating and died following long-term treatment. These results suggests that propranolol treatment promoted reduced HIF protein expression and corresponding downregulation of HIF target genes, and inhibited cell proliferation in parallel with induction of cell death by apoptosis.

**Conclusions:**

Our results suggest that propranolol could reduce the growth of HIF-dependent tumors and may thus be a promising treatment to delay surgery in VHL patients.

## Background

Von Hippel-Lindau (VHL) disease is a rare type of phacomatosis with an incidence of 1 per 36,000 individuals in the general population [1, 2]. The clinical manifestations include multiple benign and malignant tumors that appear throughout the lifespan of the patient. The most frequent tumors are hemangioblastomas (HB) of the central nervous system (CNS) and retina, and renal cell carcinoma [3–6]. In addition, pheochromocytomas, pancreatic serous cystadenoma, endolymphatic sac tumors and papillary cystadenomas are associated with the disease.

VHL disease is an inherited entity, with autosomal dominant transmission. Patients are heterozygous for mutations in *VHL*, a tumor suppressor gene located on the short arm of chromosome 3 (3p25–p26). Tumors develop in patients who have a mutated copy of *VHL* at birth following loss of the wild-type allele (loss of heterozygosity) [7]. Thus, the tumors of these patients either do not express VHL protein (pVHL) or the mutated form is not functional. pVHL binds to and ubiquitinates HIF-1α and HIF-2α to target them to the proteasome for degradation. Therefore, in the absence of functional pVHL, HIF accumulates within the cytoplasm and translocates to the nucleus to trigger the hypoxia program by targeting hypoxia responsive genes [8]. HIF-1α and HIF-2α are involved in cell proliferation, angiogenesis, extracellular matrix degradation, vascular tone, and erythropoiesis, among other processes. All HIF target genes are normally silenced in normoxia. pVHL cannot bind HIF in hypoxic conditions, since prolylhydroxylases cannot hydroxylate specific proline residues of HIF. In these circumstances, HIF accumulates and translocates to the nucleus. Therefore, cells from VHL tumors have a constitutively active HIF program due to the absence of functional pVHL.

Thus far, the therapeutic options for VHL patients are derived from surgery [9, 10]. The systemic therapy used for metastatic cancers has shown limited response in VHL pancreatic and renal tumors, while CNS tumors do not respond at all. Therefore, the lack of therapies for diffuse or recurrent disease means there is an urgent requirement for effective drugs with reduced side effects for VHL patients, especially those that halt the progression of tumors and subsequently delay surgical treatment. Some previous studies have shown that propranolol, a β-blocker used for the treatment of arrhythmia, hypertension, migraines, and other cardiac and neurological diseases, is also the best option for the treatment of infantile hemangioma (IH) [11–15]. IH is the most frequent vascular benign tumor in newborns. In the last few years, propranolol has become the choice treatment for IH over surgery, with a long list of publications supporting its success. In relation to this, our group has demonstrated that endothelial cells treated with propranolol showed decreased expression of the pro-angiogenic proteins endoglin and ALK1, which are HIF-1α targets [16]. Although the precise mechanism of action of propranolol is unclear, upon blocking β-adrenergic receptors, propranolol leads to vasoconstriction (reducing the blood flow), apoptosis induction, and inhibition of angiogenic HIF target genes such as vascular endothelial growth factor (*VEGF*), fibroblast growth factor (*FGF*) or metalloproteases (*MMP*s).

Therefore, these results led us to consider the hypothesis that propranolol could be an efficient treatment for hemangioblastomas through inhibition of HIF in highly vascularized tumors in which HIF is constitutively expressed.

## Methods

### Cell culture

HeLa 9XHRE cells were stably transfected with a HRE-luc reporter carrying nine copies in tandem of the hypoxia responsive element (HRE) followed by luciferase gene, and were cultured in DMEM (Dulbecco’s Modified Eagle Medium, Gibco, Grand Island, NY, USA) supplemented with 10 % fetal bovine serum (FBS; Gibco), 2 mM L-glutamine and 100 U/ml penicillin/streptomycin (Gibco). To induce hypoxic conditions, HeLa cells were cultured either with 100 μM desferrioxamine (DFO) (chemical hypoxia) or incubated in a hypoxic chamber (Billups-Rothemberg, Inc, Del Mar, CA) in 1 % oxygen, 5 % CO_2_ and 94 % N_2_, for 24 h.

Primary cultures of CNS hemangioblastoma were obtained according to the previously novel protocol designed by Serrano-Heras and scientific collaborators from General University Hospital of Albacete, Spain (Manuscript in preparation). Between March 2013 and march 2014 clinical samples from 4 patients (3 men and 1 woman, mean range 31.5 ± 15.6 years, and range: 13–45) diagnosed of Von Hippel-Lindau disease were taken collected from the Neurosurgery department at “Fundación Jiménez Díaz”, Universitary Hospital, Spain. Prior informed consent written forms were obtained from all patients. All procedures had been previously approved by the Ethics Committee in accordance with general accepted guidelines for human samples. The collected fresh tissue from the excess of resected hemangioblastoma was placed in sterilized tubes containing ice-cold Earle’s Balanced Salt Solution (EBSS, Gibco). Tumor samples were washed several times with PBS, and cut into 1 mm3 pieces. Tissue pieces were transferred with sterile tweezers to clean cell culture dishes (Sarstedt, Germany) and subjected to enzymatic digestion with equal amount of collagenase I and dispase II to a final concentration of 1 mg/m in EBSS, for 45 min at 37 °C. After that, the pieces were completely disaggregated by gently pipetting and digested for 15 min with trypsin at 37 °C. Then, minced samples were centrifuged, cell pellets were suspended in growth medium (RPMI 1640 (Gibco) supplemented with 20 % fetal bovine serum, 1 % Pen/Strep and 4 mM of glutamine), and incubated at 37 °C. Medium was replaced every 72 h until the cultures were confluent.

Furthermore, Flow Cytometry was performed for cell characterization. The analysis showed that cultures were composed of stromal cells (30–50 % CD99 + cells), endothelial cells (15–25 % CD34+ cells), and pericytes (30–40 % NG2+ cells). The experiments were performed in all types of cells composing the hemangioblastoma. A total of four hemangioblastomas from different patients were assayed in culture, with the following localization: HB2, spinal bulb; HB3, temporal lobe; HB7, spinal cord; HB11, temporal lobe.

### Real-time RT-PCR

Total cellular RNA was extracted from hemangioblastoma cells using a Nucleo Spin RNA kit (Macherey-Nagel, Düren, Germany). One microgram of total RNA was reverse-transcribed in a final volume of 20 μl with the First Strand cDNA Synthesis Kit (Roche, Mannheim, Germany) using random primers. SYBR Green PCR system (BioRad, Hercules, CA, USA) was used to carry out real-time PCR with an iQ5 system. The sequences of the oligonucleotides used corresponded to HIF-1α and -2α target genes, as follows: *VEGF* forward: 5’-ATCTGAGCAGGGCGACAGC-3’ and reverse 5’-ACTCCCTGTGGTGCAGTCA-3’; *EPO* forward 5’-TGTTTTCGCACCTACCATCA-3’ and reverse 5’-AAGTCACAGCTTGCCACCT-3’; and *SOX2* forward 5’-GGGGGAATGGACCTTGTATAG-3’ and reverse 5’-CGCTCCACCAACTAAGAACG-3’. As an internal control, mRNA levels of *18S* were measured using the following primers: forward 5’-CTCAACACGGGAAACCTCAC-3’ and reverse 5’-CGCTCCACCAACTAAGAACG-3’. Amplicons were detected using an iQ5 system (BioRad). The samples were used in triplicate and the experiment was repeated twice.

### Western blot analysis

Cells were lysed on ice for 30 min in TNE buffer (Tris 50 mM NaCl 150 mM-EDTA 1 mM 0.5 % Triton X100) supplemented with protease inhibitors (Complete Roche Diagnostics) and lactacystin as a specific proteasome inhibitor to preserve HIF. Lysates were centrifuged at 14,000 × *g* for 5 min. Similar amounts of proteins from aliquots of cleared cell lysates were boiled in SDS sample buffer and analyzed by 10 % SDS-PAGE under non-reducing conditions. Proteins from gels were electrotransferred to nitrocellulose membranes followed by immunodetection with anti-HIF1α (BD, Bedford, MA, USA), anti-HIF-2α (NOVUS, Oxon, UK) and anti-γ-tubulin (Sigma, St.Louis, MO, USA) antibodies at the dilution recommended by the manufacturer. Secondary antibodies were horseradish peroxidase conjugates from Dako (Glostrup, Denmark). Membranes were developed by chemiluminescence (SuperSignal West Pico Chemiluminescent Substrate, Thermo Scientific, Rockford, IL, USA).

### Luminescent cell viability assay

The viability of hemangioblastoma and HeLa 9X HRE cells was measured with a CellTiter-Glo Luminescent Cell Viability Assay (Promega, Madison, WI, USA). This is a homogeneous method to determine the number of viable cells in culture based on quantitation of the ATP present, which signals the presence of metabolically active cells. A total of 10,000 cells were plated in 96-well plates and cultured for 24 h and 48 h with propranolol (50 μM and 100 μM) in 100 μl of medium. After treatment, plates were equilibrated to room temperature for 30 min before the addition of 100 μl of Cell Titer-Glo reagent (Lysis buffer, Ultra-Glo Recombinant Luciferase, Luciferine and Mg^2+^). Cell lysis was induced in an orbital shaker for 2 min, and then plates were incubated at room temperature for 10 min to stabilize the luminescent signal. Luminescence was measured using a Glomax Multidetection System (Promega).

### Caspase activation assay

The Caspase-Glo 3/7 Assay (Promega) is a luminescent assay that measures caspase-3 and caspase-7 activity using a luminogenic caspase-3/7 substrate that contains the tetrapeptide sequence DEVD in a reagent optimized for caspase activity, luciferase activity and cell lysis. Luminescence is proportional to the amount of caspase activity present. A total of 10,000 hemangioblastoma cells were plated in 96-well plates and cultured for 24 h and 48 h with propranolol (50 μM and 100 μM) in 100 μl of medium. After treatment, plates were equilibrated to room temperature for 30 min before the addition of 100 μl of Caspase Glo 3/7 Reagent (lysis buffer, Ultra-Glo Recombinant Luciferase, DEVD-aminoluciferine and Mg^2+^). Cell lysis was induced by shaking for 30 s (300 rpm), and then plates were incubated at room temperature for 2 h. Luminescence was measured using a Glomax Multidetection System (Promega).

### VEGF determination in plasma

A Quantikine Human VEGF ELISA kit from R&D Systems (Abingdon, UK) was used to quantitatively determine human VEGF-A concentration in supernatants of hemangioblastoma culture cells treated with propranolol at different doses.

### Statistics

Data represent mean ± SD. Differences in mean values were analyzed using the Student’s *t*-test. *P*-values of <0.05 were considered to be statistically significant; statistically significant values are marked with asterisks (*P < 0.05; **P < 0.01; ***P < 0.005).

## Results

### Propranolol downregulates HIF-dependent transcription in 9XHRE HeLa cells

**Fig. 1:**
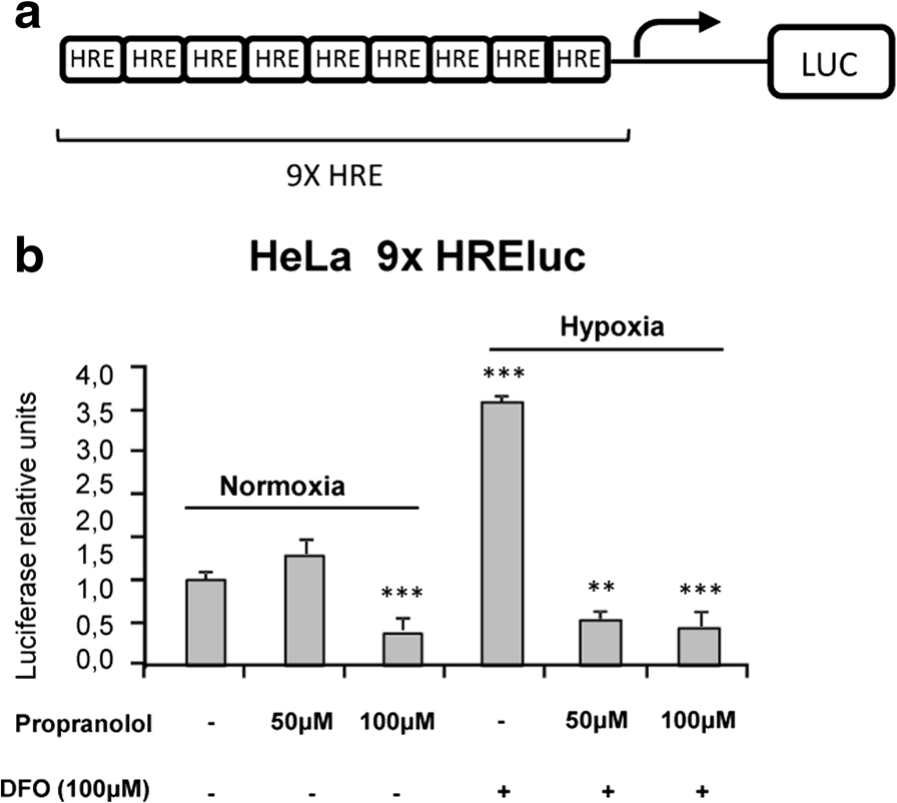
Propranolol downregulates HIF-dependent transcription in HeLa cells. **a** Schematic structure of 9XHRE-luc reporter. **b** HIF-dependent transcription in HeLa reporter cell line. Luciferase assays were performed in HRE-luc-stably transfected HeLa cells under hypoxic conditions and propranolol treatment. Propranolol (50–100 μM) prevented hypoxia stimulation in HeLa cells, as shown by the decrease in luciferase activity, by inhibiting the activation of hypoxia elements (HRE) by HIF. ***P < 0.005

HeLa cells stably transfected with HRE-luc reporter were used to measure HIF-dependent transcription (Fig. 1a). Hypoxia activation was measured quantitatively by a luminometry assay under hypoxic conditions. When HeLa cells were subjected to chemical hypoxia (100 μM DFO), a marked increase in luciferase units, over normoxic conditions, of up to 3.5 fold was detected. However, treatment of the cells in hypoxic conditions with 50 and 100 μM propranolol counteracted the hypoxic stimulation of HeLa 9XHRE cells, as shown by a decrease in luciferase activity (Fig. 1b). The results were statistically significant in all cases, with the exception of those obtained under normoxia and for treatment with 50 μM propranolol (*P* < 0.005). Culture of cells in a hypoxic chamber (1 % O_2_) with or without propranolol yielded essentially the same results as with DFO (data not shown).

### Propranolol decreases the expression of HIF target genes in hemangioblastoma cells

**Fig. 2:**
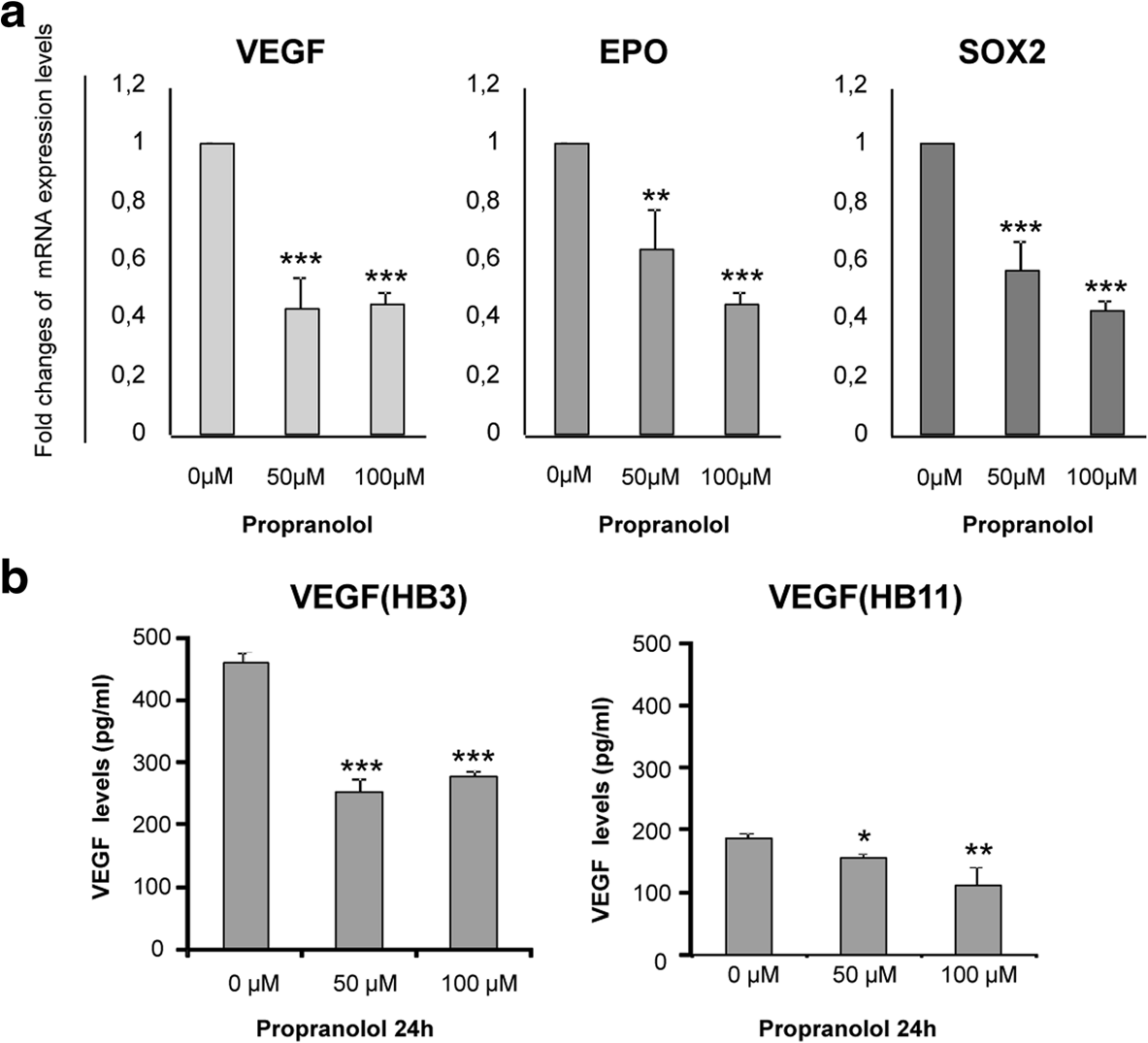
Propranolol decreases the expression of HIF target genes in hemangioblastoma cells. **a** The transcription levels of HIF target genes, *VEGF*, *EPO* and *SOX2*, were compared with the endogenous control of 18S ribosomal RNA. Propranolol treatment led to a dose-dependent decrease in mRNA expression as a consequence of the reduced expression of HIF targets. Differences were statistically significant according to the Student’s *t*-test. *P < 0.05; **P < 0.01; ***P < 0.005. **b** VEGF soluble levels were measured by ELISA assay in supernatants of hemangioblastoma cultures treated with propranolol. Soluble VEGF levels were decreased in propranolol-treated cells compared with untreated ones

Next, we explored the effects of propranolol treatment of hemangioblastoma cells over HIF targets. Thus, the expression of genes transcriptionally regulated by HIF-1α, such as *VEGF* and *EPO* (erythropoietin), and HIF-2α, such as *SOX2* (SRY-related HMG BOX) was studied by quantitative RT-PCR. As shown in Fig. 2a, the levels of transcripts of all these genes were significantly decreased by 40–60 % from a dose of 50 μM. The levels of *EPO* and *SOX2* were reduced in a dose-dependent manner; at 100 μM propranolol, the level of the different mRNAs was about 40 % of the controls. These results were observed in all four hemangioblastomas samples. The results agree with a lower transcription level of HIF target genes. Notably, *VEGF* and *EPO* are HIF-1α targets involved in angiogenesis and oxygen supply to cells, while *SOX2* is a transcriptional target of HIF-2α and is a stemness target implicated in the undifferentiated nature of the mesenchymal component predominant in hemangioblastomas [17-19].

*VEGF*, one of the main targets of HIF-1α, and a potent mediator of both angiogenesis and vasculogenesis, was also measured in the supernatants of two different hemangioblastoma samples cultured with and without propranolol. As observed in Fig. 2b, the levels of *VEGF* were decreased by treatment with 50 μM propranolol, in agreement with the mRNA results (Fig. 2a).

### Propranolol affects the viability of tumor cells and induces cell death by apoptosis

**Fig. 3:**
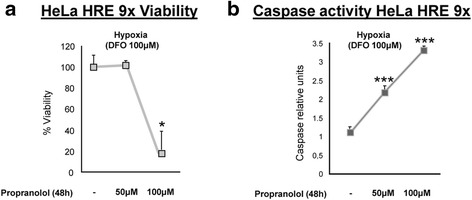
Propranolol affects viability and caspase activation in HeLa cells. HeLa 9XHRE cells were cultured in the absence or presence of propranolol (50 μM or 100 μM). Viability was measured using a CellTiter-Glo assay (**a**), and apoptosis was measured using a Caspase-Glo 3/7 assay (**b**). The histograms show an increase in the activation of caspase 3/7 and a reduction in cell viability

Albiñana et al. (2012) reported that propranolol induces apoptosis and decreases viability of human endothelial and EOMA cells (murine hemangioendothelioma endothelial cells) [16, 20]. Viability of HeLa cells after DFO treatment (chemical hypoxia), with and without propranolol, was quantitatively measured using the CellTiter-Glo luminescent cell viability assay. As shown in Fig. 3a, propranolol treatment affected the viability of HeLa cells. On the other hand, when apoptosis was measured quantitatively with the Caspase-Glo 3/7 luminescent assay, it was found that propranolol reduces viability of HeLa cells through an increase in apoptosis by caspase 3/7 activation. After 48 h propranolol treatment, cells stopped proliferating, and the increase in caspase activity promoted cell death (Fig. 3b).

**Fig. 4:**
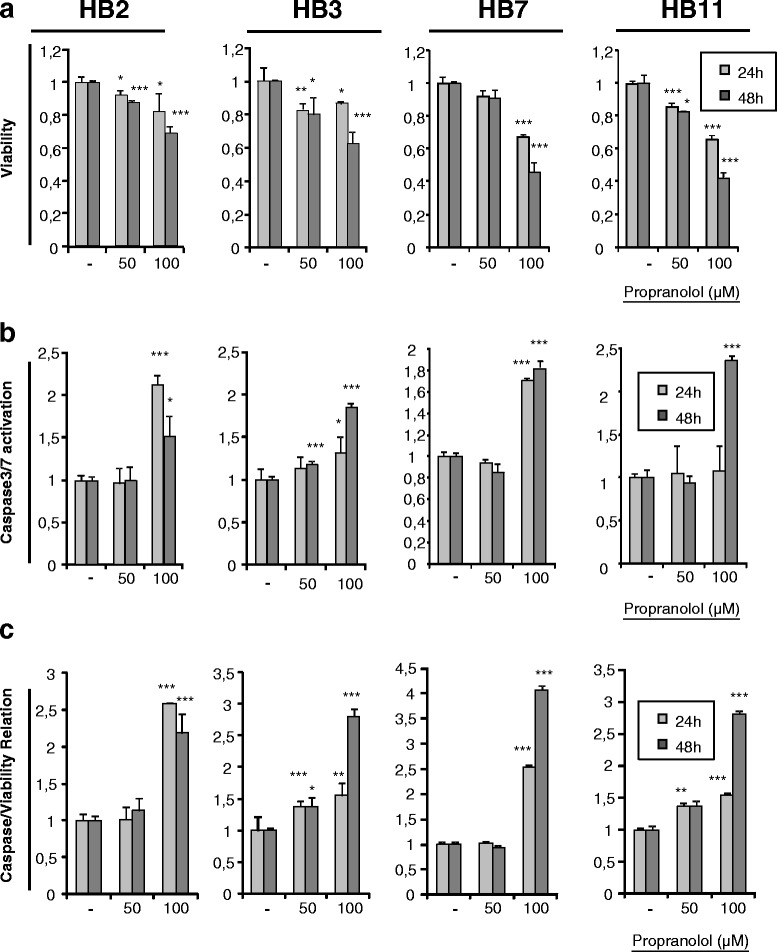
Propranolol affects viability and caspase activation in hemangioblastoma cells. Different lines of hemangioblastoma cells were cultured in the absence or presence of propranolol (50 μM or 100 μM) to measure viability and apoptosis. The histograms show a decrease in viability (**a**), with a parallel increase in the activation of caspase 3/7 (**b**). Finally, in relation to the decreased cell viability, the ratio of apoptosis *vs.* viability is shown (**c**). Propranolol promoted a decrease in viability by increasing apoptosis in these tumor cells

Next, we used the same assays to check survival and apoptosis of hemangioblastoma cells after propranolol treatment for 24 and 48 h. Figure 4 shows viability, caspase 3/7 activation, and the caspase activation/viability ratio in the four hemangioblastoma cultures from four different VHL patients; viability decreased with propranolol in a concentration- and time-dependent manner in almost all cases. Here, each hemangioblastoma represents a different culture from a different patient and different tumor, therefore there is wide variability, but we show in all cases that the response is qualitatively similar. The lowest viabilities were found after treatment with 100 μM of propranolol for 48 h. This decrease in viability can be explained by an increase in apoptosis through caspase 3/7 activation, particularly at 100 μM and after 48 h of treatment.

**Fig. 5:**
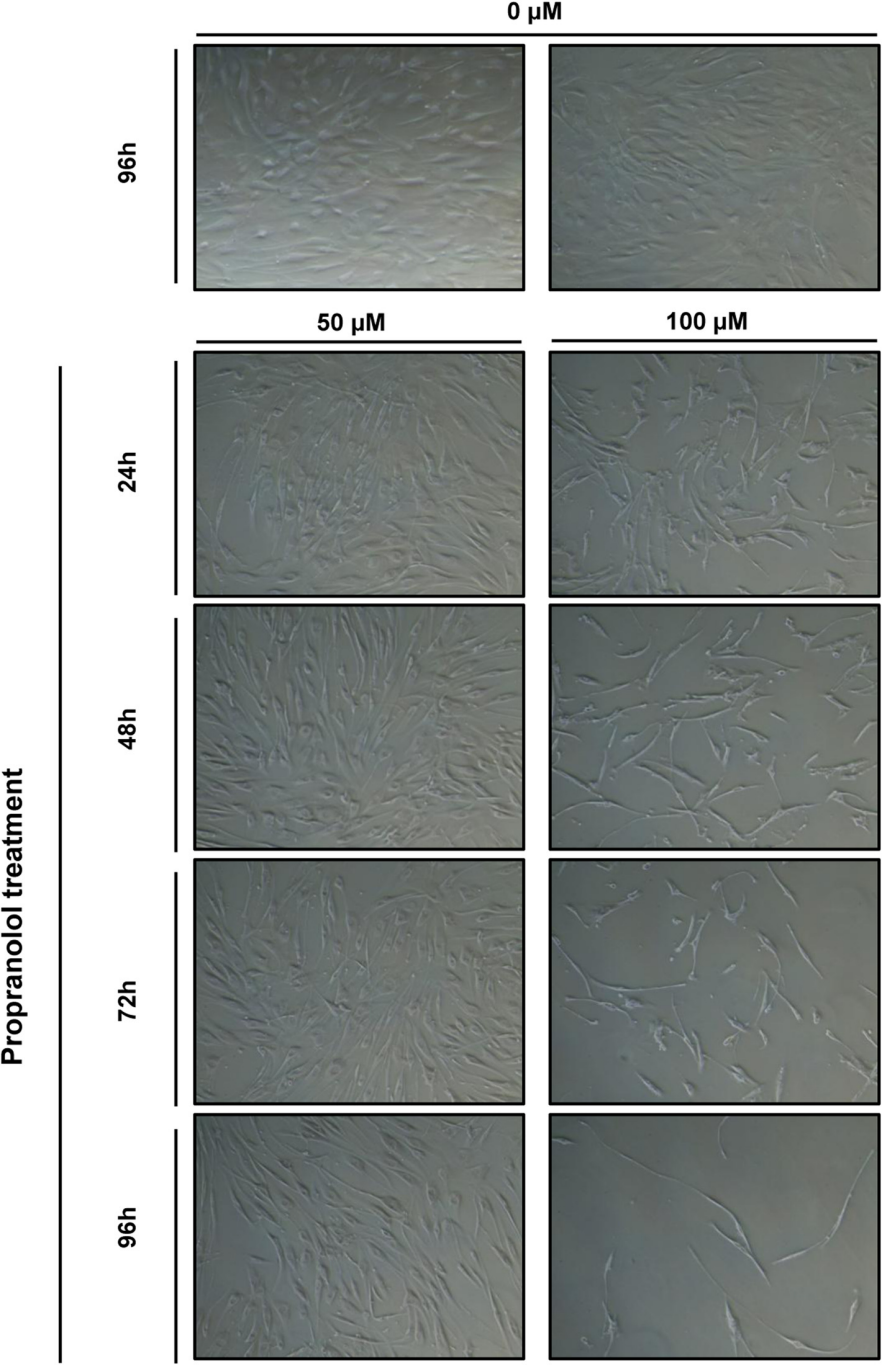
Long-term propranolol treatment inhibits proliferation and promotes death of hemangioblastoma cells. Time course of propranolol treatment. Hemangioblastoma cells were treated at different doses (50 μM and 100 μM) and for varying lengths of time (from 24 h to 96 h) with propranolol. Significant changes in morphology in treated cells were observed at 100 μM, while at 50 μM the cells exhibited the same morphology as the control cells but with characteristic nucleoli. After 96 h with 100 μM treatment, most cells had disappeared from the plate as a consequence of cell death by apoptosis (Magnification 40X)

Given the previous results of viability reduction and apoptosis induction after propranolol treatment, we decided to subject hemangioblastoma cells to long-term propranolol treatment, starting with 50,000 cells per well, and recording images in a time course follow-up. As shown in Fig. 5, hemangioblastoma cells appeared to stop proliferating, and then an increase in cell death is detected since there were empty spaces in the plates. The death, as explained by the previous results (Fig. 4), must be attributed to apoptosis. After 5 days (96 h) of continuous propranolol treatment, there were few cells remaining in the 100 μM propranolol-treated cultures, with fewer than 5,000 cells with poor morphology, compared with 300,000 healthy cells in the untreated cultures. The remaining propranolol-treated cells, on the other hand, exhibited an atypical and apoptotic appearance.

Altogether, we can conclude that propranolol decreases the viability of tumor cells (HeLa and VHL-derived hemangioblastoma cells) by stopping proliferation and inducing cell death by apoptosis. This result would be compatible with the regression observed in IH, and it would suggest that propranolol could delay the proliferation of hemangioblastomas in VHL patients.

**Fig. 6:**
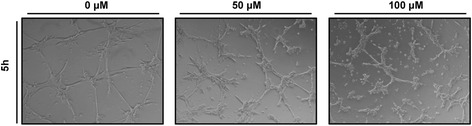
Propranolol inhibits angiogenesis. Treatment with propranolol inhibits tubulogenesis of hemangioblastoma cells partially (50 μM) or completely (100 μM). (Magnification, 40X)

Hemangioblastomas have a heterogeneous cell composition consisting of pericytes, stromal and endothelial cells. Propranolol affects all cell types. As a complementary result, Fig. 6 also supports the loss of migration and angiogenic activity of hemangioblastoma cells after propranolol treatment. In fact, propranolol treatment prevents the tubulogenesis displayed by the hemangioblastoma cells in matrigel. The endothelial/pericyte cells of hemangioblastomas display a typical network arrangement in special culture conditions such as matrigel. Thus, hemangioblastoma cells without propranolol treatment tend to build “tube-like structures of cells”, while propranolol abrogates this manifestation of *in vitro* angiogenesis.

## Discussion

**Fig. 7:**
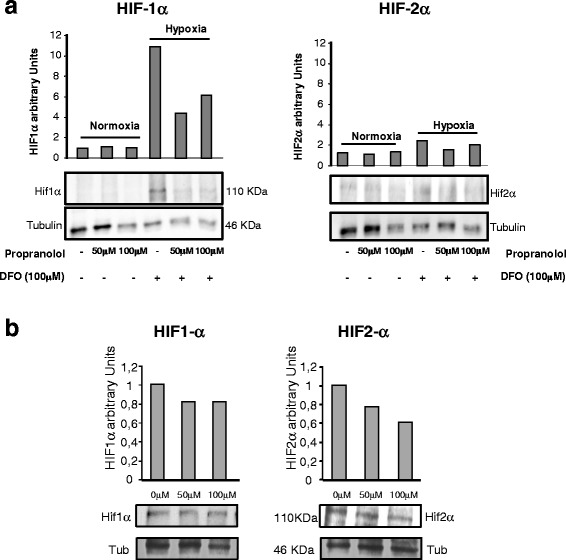
HIF-1α and HIF-2α protein expression is decreased following propranolol treatment. **a** Western blot assays were performed on lysates from 9XHRE HeLa cells. In agreement with luciferase assays, there were no differences in HIF-1α and HIF-2α protein levels between the different propranolol treatments under normoxia. After DFO treatment (simulating hypoxia), levels of both proteins increased. When propranolol was added to the cells, HIF-1α and HIF-2α levels decreased. **b** Western blot analysis was performed on lysates from hemangioblastoma cells treated with 50 and 100 μM propranolol for 24 h; HIF-1α and HIF-2α levels were reduced. γ-Tubulin was used as loading control

Von Hippel-Lindau (VHL) is an autosomal dominant disease that results in many highly vascularized tumors disseminated over the body. VHL is an age-dependent and highly penetrant disease, and the more common manifestations include retinal and CNS hemangioblastomas, clear cell renal carcinomas, pheochromocytomas, and endolymphatic sac tumors of the middle ear [21, 22]. The mechanism for the pathogenesis of VHL disease is explained by the two-hit hypothesis [23]. One allele of *VHL* is usually an inherited mutant copy while the second hit is acquired through somatic mutation or hypermethylation. Tissue microdissection has showed *VHL* gene deletion in the stromal cells of hemangioblastomas [24], indicating that stromal cells are the true tumor cells derived from embryologically arrested hemangioblasts that are able to develop into hematopoietic and endothelial progenies under suitable conditions [2]. VHL is a complicated systemic disease with serious involvement throughout the entire body. Currently, there is no cure, nor successful therapeutic options, except surgery to remove the VHL tumors. Therefore, there is a need for drugs to target tumour growth to delay or remove the need for surgery. VHL tumors express HIF1α and HIF2α in a constitutive manner [17, 18] since they lack functional pVHL. HIF is a transcription factor that can activate hundreds of target genes involved in angiogenesis, tumor formation, survival, invasion, and metabolism [25–27]. Given that tumors from VHL patients lack functional pVHL, HIF is constitutively expressed in normoxic conditions, as corroborated by the high levels of HIF detected in these tumors by western blotting (Fig. 7). This is in contrast with HeLa cells under normoxic conditions where there is no HIF protein, unless subjected to hypoxic conditions, as previously reported [28]. Hence, a drug targeting HIF, and thus the genes it regulates, would be the ideal strategy to delay VHL tumor progression.

It has been suggested that propranolol may act through different ways, including vasoconstriction, as an anti-angiogenic inhibiting VEGF production, and as a pro-apoptotic drug leading to cell apoptosis [11, 20, 29, 30]. However, its potential therapeutic benefits in different vascular anomalies remain to be explored.

Based on previous results of propranolol efficiency for IH treatment [12–15], and our own results showing that propranolol acts as antiangiogenic in endothelial cells by decreasing transcription levels of proangiogenic factors such as endoglin and ALK1 [16], we hypothesized that propranolol could act by decreasing HIF levels and therefore downregulating the HIF target program. As a putative side effect of propranolol treatment, concerns about long-term neurodevelopmental or cognitive effects of propranolol have recently arisen after a study by Langley and Pope [31]. These authors argue that propranolol can cross the blood–brain barrier and can cause sleep and memory disturbances, as demonstrated by decreased specific memory functions in adults. This work was commented upon by Tozzi [32] in a letter to the Editor, and Léauté-Labrèze et al. responded [33] in the same issue of the journal. The reply is supported, first of all, by the lack of adverse events in children treated with propranolol. In fact, the rates of neurodevelopmental defects or delay observed among 272 propranolol-treated children from 2008 to 2013 seem to be within the range observed in the normal population. On the other hand, the article by Langley and Pope is essentially theoretical and not evidence-driven, and includes data from small studies in healthy adults.

Interestingly enough, all the HIF target genes, including among others, *VEGF*, *MMPs*, *EPO* or *FGF*, are absolutely necessary for the survival and progression of tumors in general, and for hemangioblastomas in particular. Hemangioblastomas are complex tumors consisting of different cellular types, with stromal (undifferentiated mesenchymal cells) and endothelial cells as the main components. Thus being so, propranolol might be able to stop vascular dependent growth of hemangioblastomas [2, 34].

In the present work, we have shown that propranolol downregulates HIF-1 targets. In fact, propranolol inhibited HIF-dependent transcription in a 9xHRE HeLa reporter cell line (Fig. 1) and the crucial gene targets for hemangioblastoma survival, such as *VEGF*, *EPO* and *SOX2* (Fig. 2). EPO has been reported to be active in hemangioblastoma tumorlets of VHL patient [35–37], and according to Fig. 2a its expression was reduced by almost half in the presence of propranolol. On the other hand, VEGF was shown to be downregulated at the transcriptional level (Fig. 2a), but also as protein secreted into the culture supernatants of different hemangioblastomas cultivated *in vitro* (Fig. 2b). Moreover, the impaired angiogenesis of hemangioblastomas in matrigel is shown in Fig. 6, where the tubular network of untreated cells remained with unclosed cells at an intermediate dose of propranolol, and its formation was prevented at the highest propranolol dose. This is in agreement with the results obtained by Albiñana et al. (2012) where propranolol precluded the tube formation of treating EOMA and human endothelial cells at the highest dose.

Hemangioblastoma tissue is composed of neoplastic stromal cells and abundant reactive vascular cells [19, 24, 38]. VHL-deficient tumor cells have hemangioblastic differentiation capacity [39, 40]. Stromal cells are undifferentiated cells of mesenchymal nature, where SOX-2 master stemness marker is expressed as a HIF-2α target. Thus, we explored the expression of SOX-2 in hemangioblastoma before and after propranolol treatment, and SOX-2 levels were also significantly decreased by propranolol (Fig. 2).

We established that propranolol decreased HIF-dependent transcription in 9XHRE HeLa cells under hypoxia and in hemangioblastoma cells constitutively expressing HIF, as shown in Fig. 7a, the amounts of HIF1α and HIF2α protein under hypoxic conditions in HeLa cells were decreased by propranolol treatment. This fact was also tested in hemangioblastoma cells that were functionally hypoxic, as shown by the expression of HIF1α and HIF2α under normal culture conditions. Western blotting demonstrated that both HIF-1α and 2α were decreased by propranolol treatment at the protein level (Fig. B).

Next, we explored whether propranolol could interfere with proliferation and survival of hemangioblastoma cells. As HIF triggers the hypoxia gene program to maintain cell survival, the previous results suggested that propranolol also affected cell viability. In fact, Fig. 3 demonstrates that propranolol reduced the viability of HeLa cells under hypoxic conditions. The cause of death was determined to be apoptosis, as measured by the activity of caspases 3/7, which was increased by three-fold over untreated cells at the highest dose of propranolol. Interestingly, the same holds true when the viability and apoptosis of hemangioblastoma cells from different VHL patients were evaluated after propranolol treatment. Figure 4 shows that hemangioblastoma cell viability may drop by up to 50 % after 48 h, and that apoptosis is increased between 2.5- and 5-fold following 48 h of 100 μM propranolol treatment.

Given these results, our assumption was that, if propranolol treatment was sustained in time, cells would disappear as is shown in Fig. 5 in hemangioblastoma cells. Notably, after 96 h at 100 μM propranolol treatment, very few cells remained attached to the wells, and those that remained alive had an extremely thin and elongated shape, showing the appearance of apoptotic cells.

**Fig. 8:**
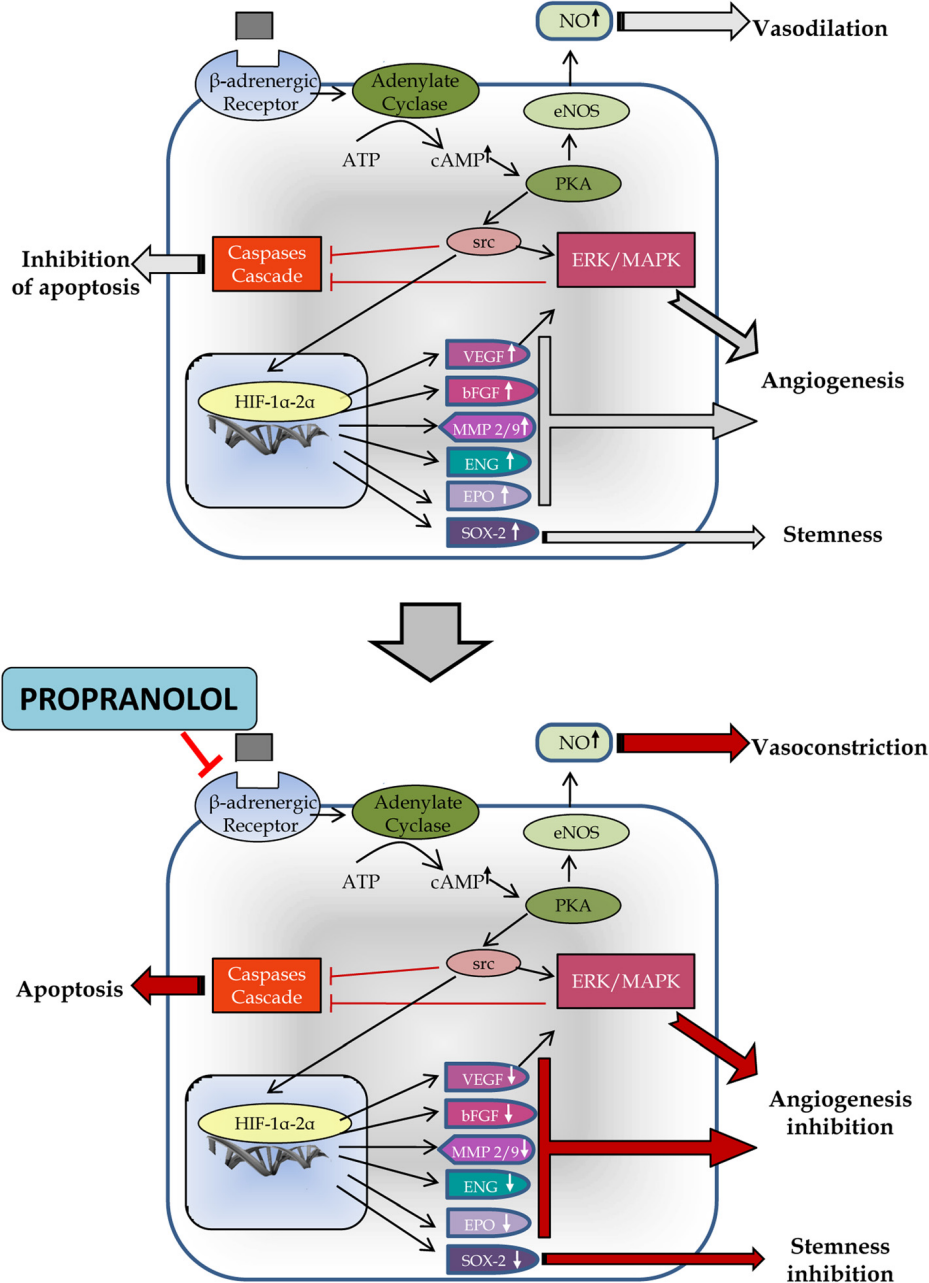
Hypothetical model of propranolol mechanism of action

Altogether, the results suggest that propranolol first decreases HIF levels in hemangioblastoma cells, and in this way, the HIF targets are partially silenced/decreased. Consequently, in the absence of essential factors for survival, the tumor cells, stop dividing and die by apoptosis. A scheme showing the hypothetical mechanism of action of propranolol investigated in the present paper is presented in Fig. 8.

## Conclusions

In conclusion, our results suggest that propranolol reduces the growth of HIF-dependent tumors, and may be a promising therapeutic drug to delay surgery in VHL patients.

## Abbreviations

ALK1: Activin receptor like kinase 1
CNS: Central nervous system
DFO: Deferroxiamine
DMEM: Dulbecco’s modified Eagle medium
EOMA: Mouse hemangioendothelioma edothelial cells
EPO: Erythropoietin
FBS: Fetal bovine serum
FGF: Fibroblast growth factor
HIF: Hypoxia inducible factor
HRE: Hypoxia responsive element
IH: Infantile hemangioma
MMP: Matrix metalloproteinases
pVHL: Von Hippel Lindau protein
RPMI: Roswell Park Memorial Institute
RT-PCR: Reverse transcription polymerase chain reaction
SDS-PAGE: Sodium dodecyl sulfate-polyacrylamide gel electrophoresis
SOX-2 or SRY-box 2: Sex-determining region Y-box
VEGF: Vascular endothelial growth factor
VHL: Von Hippel-Lindau
